# Glucans: A Therapeutic Alternative for Sepsis Treatment

**DOI:** 10.1155/2024/6876247

**Published:** 2024-05-27

**Authors:** Jesse P. M. Viana, Fernanda F. Costa, Tatielle G. Dias, Priscila M. Mendes, Gabriel B. Copeland, Willian S. Nascimento, Sofia S. N. Mendes, Isabella F. S. Figueiredo, Elizabeth S. Fernandes, Anamelia L. Bocca, Márcia C. G. Maciel

**Affiliations:** ^1^ Departamento de Biologia Celular Instituto de Ciências Biológicas Programa de Pós Graduação em Ciências Biológicas (Biologia Molecular) Laboratório de Imunologia Aplicada Universidade de Brasília (UnB), Brasília, Brazil; ^2^ Programa de Pós-graduação em Saúde e Tecnologia Universidade Federal do Maranhão, São Luís, Brazil; ^3^ Programa de Pós-graduação em Ciências da Saúde Universidade Federal do Maranhão, São Luís, Brazil; ^4^ Laboratório de Imunologia Aplicada Universidade de Brasília (UnB), Brasília, Brazil; ^5^ Programa de Pós-graduação em Biotecnologia Aplicada à Saúde da Criança e do Adolescente—Faculdades Pequeno Príncipe Instituto de Pesquisa Pelé Pequeno Príncipe, Curitiba, Brazil; ^6^ Plataforma Bi-Institucional de Pesquisa Translacional—Fiocruz/SP, São Paulo, Brazil

## Abstract

Sepsis treatment is a challenging condition due to its complexity, which involves host inflammatory responses to a severe and potentially fatal infection, associated with organ dysfunction. The aim of this study was to analyze the scientific literature on the immunomodulatory effects of glucans in a murine model of systemic infection induced by cecal ligation and puncture. This study comprises an integrative literature review based on systematic steps, with searches carried out in the PubMed, ScienceDirect, Scopus, Web of Science, and Embase databases. In most studies, the main type of glucan investigated was *β*-glucan, at 50 mg/kg, and a reduction of inflammatory responses was identified, minimizing the occurrence of tissue damage leading to increased animal survival. Based on the data obtained and discussed in this review, glucans represent a promising biotechnological alternative to modulate the immune response and could potentially be used in the clinical management of septic individuals.

## 1. Introduction

Sepsis is a global public health problem, causing an estimated 11 million deaths annually [1]. Despite a decrease in its incidence and mortality rates, sepsis remains one of the main contributors to decline in health on a worldwide scale, affecting almost 50 million individuals globally [2]. Sepsis can develop from bacterial, viral, fungal, or parasitic infections [3], and it is characterized by a deregulated inflammatory response to an infection, culminating in multiple organ dysfunctions. Currently, the most widely used therapeutic interventions for sepsis aim to controlling the infectious agent through the administration of antimicrobials, combined with supportive approaches, such as fluid resuscitation. Individual immunomodulatory treatments have been used and were able to reduce mortality in some but not all patients including IFN-*γ*, GM-CSF, IL-7, PD-1 antagonists, cannabinoids, thymosin *α*-1, PMX-HP, IL-6 inhibitors, and IL-1*β* antagonists [4]. Thus, there is a constant search for immunomodulators motivated by the lack of specific drugs to treat patients with sepsis. In this context, investigations of new therapeutic strategies, such as *β*-glucan, a natural compound that is abundant in nature and has immunomodulatory properties, remain a focus [5, 6].

Glucans are a group of natural polysaccharides with wide structural diversity, found in various sources, such as cereals, mushrooms, and yeasts. Their consumption has been linked to anticancer [7], antidiabetic [8], and immune system stimulatory [9] activities, as well as to the treatment of infections [10] and sepsis [11]. *β*-Glucans are structurally described as D-glucose polymers joined via *β*-glycosidic bonds [12]. Despite the diversity of available sources for the extraction of *β*-glucans, the most widely investigated are the fruiting bodies of different types of mushrooms, notably yielding (1 → 3) and (1 → 6)-*β*-glucans [13]. The immunomodulatory activity of glucans occurs mainly through the stimulation of immune cells *via* pattern recognition receptors (PRRs), such as Toll-like receptors (TLRs), complement receptor-3 (C3), and receptor for glucans on dendritic cells (dectin-1) [14].

Dectin-1 is the most well-studied receptor for *β*-glucans, originally described as the “*β*-glucan receptor expressed on myeloid cells, playing essential roles in immune responses against fungi” [15]. It is a glycoprotein that has a carbohydrate recognition domain, allowing it to identify glucans [16]. This receptor is mainly expressed on cells of myeloid origin, such as dendritic cells, macrophages, and neutrophils, although its expression has been also observed on cells of lymphoid origin, such as T and B lymphocytes [17]. Ni et al. [18] demonstrated that the interaction of a monoclonal antibody (mAb) anti-dectin-1 with dectin-1 receptors induces dendritic cells to stimulate CD8^+^ T lymphocyte responses. As a result of this interaction, a positive regulation of costimulatory molecules and secretion of cytokines and chemokines, leading to increased antigen presentation, activation, and expansion of CD8^+^ T cells, were observed [14]. Similar effects on CD8^+^ T cells were attributed to *β*-glucans [19].

Although the biological effects of *β*-glucan exposure are not yet fully understood, the literature suggests these polysaccharides are strong stimulators of the immune system. Recently, *β*-glucans were found to have the ability to induce trained immunity. Indeed, this group of molecules can stimulate innate immune responses by acting on cells such as neutrophils, monocytes, and macrophages, resulting in reprogramming of their metabolic and epigenetic states. Once the body is exposed to a second stimulus with *β*-glucans or other infectious challenges, an amplified innate immune response results, translating into the enhanced recruitment of immune system cells. This contributes to a more effective eradication of invading pathogens and consequently improves the ability of the body to fight the infection and survive [5].

In summary, the study of glucans has become increasingly important, especially because of their immunomodulatory effects. Research has demonstrated their protective properties against the lung damage caused by sepsis, evidenced by a reduction in the percentages of neutrophils and lymphocytes in the bronchoalveolar lavage [20], and increased survival rates in septic animals [21].

The aim of this integrative review is to describe the immunomodulatory potential of glucan treatment by focusing in a murine model of sepsis induced by cecal ligation and puncture. At the end of this review, we provide a comprehensive understanding of the biotechnological potential of glucans from a clinical perspective in order to provide with information for both healthcare professionals treating patients with sepsis and researchers seeking better therapeutic approaches.

## 2. Materials and Methods

An integrative literature review was developed to provide an extensive analysis of the potential of glucans to treat sepsis. The steps followed to construct this review were as follows: identification of the topic and elaboration of the guiding research question; selection and extraction of data; categorization of studies; data analysis; synthesis of results; and presentation of the integrative review [22]. We developed the research strategy following the PICO strategy, using the guidelines of the Joanna Briggs Institute (JBI), an acronym where “P” represents the population (glucan), “I” the interest (cecal ligation and puncture), and “CO” the context (sepsis). We thus obtained the following guiding question, from which we selected keywords for the database search: “What is the scientific evidence related to the effects of glucans in a model of cecal ligation and puncture in septic conditions?”

To find relevant studies, five databases were searched, PubMed, ScienceDirect, Scopus, Web of Science, and Embase; these were chosen because of their wide coverage and importance in the research area. The searches were conducted using keywords and descriptors controlled by Medical Subject Headings (MeSH) and the noncontrolled terms “glucan,” “sepsis,” and “cecal ligation and puncture,” along with the Boolean operators “AND” and “OR,” adapting them to each specific database when necessary.

The Rayyan platform was used to organize the articles obtained and to help excluding duplicated articles and reviews [23]. The inclusion criteria considered only studies that addressed the effects of *β*-glucans in sepsis models of cecal ligation and puncture. Studies published in English and available in full were included. Articles that did not include all the proposed descriptors were duplicates and narrative, integrative, or systematic reviews, and meta-analyses were excluded from the current review, as well as theses, dissertations, and unavailable articles. We used an adaptation of the Preferred Reporting Items for Systematic Reviews and Meta-Analyses (PRISMA) flowchart to describe the stages of article selection [24]. The data were collected based on the following information: year of publication, title, objective, type of study, type of glucan, dose, route of administration, treatment schedule, and conclusions. After extracting the data, the immunomodulatory and therapeutic potential of *β*-glucans was considered for a clearer understanding of the topic.

## 3. Results

**Figure 1:**
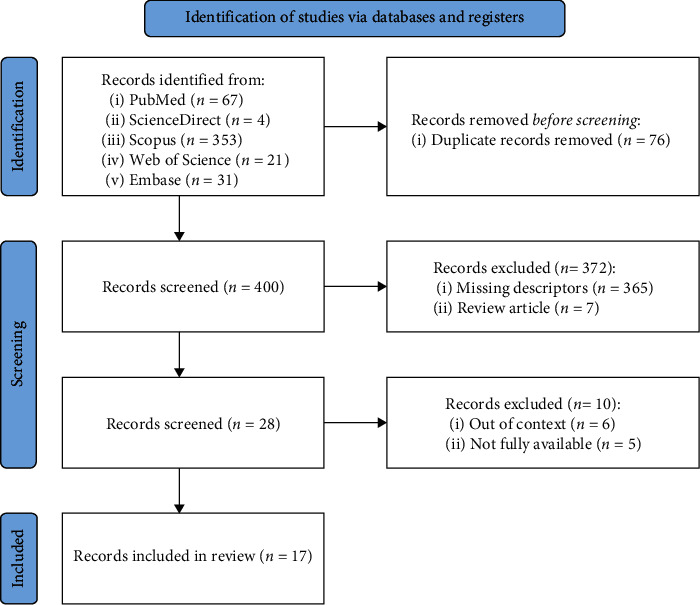
Article selection flowchart (adapted from PRISMA 2020 flowchart).

Following the searches in the databases, 476 studies were identified. Of these, 76 duplicates were excluded, leaving 400 studies. Then, 372 studies were excluded either for having missing descriptors or being review articles. After screening, 28 studies were selected to be read in full, with 11 of them being removed for being either out of context or unavailable in full. Therefore, 17 articles were included in this review (Figure 1).

**Figure 2:**
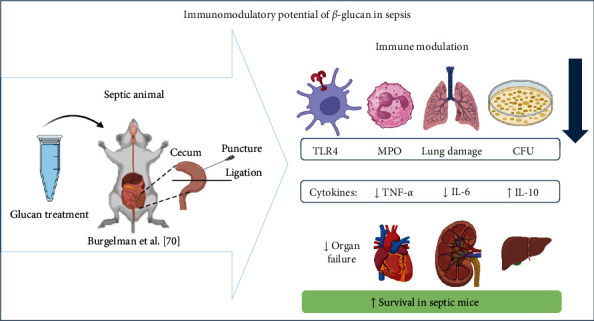
Mechanism underlying the protective effects of glucan treatment in cecal ligation and puncture (CLP)-induced infections. Prophylactic and/or therapeutic treatment enables a better immune response from the host in the fight against infection. The mechanism involved in the response involves the modulation of the expression of genes related to mortality, such as TLR-4. The treatment made it possible to reduce the expression of these genes, as well as related proteins, correlating with improved long-term survival. In addition, it allows control of colony-forming units and facilitates a reduction in plasma levels of enzymes that actively participate in the inflammatory process, such as myeloperoxidase (MPO), which is strongly associated with neutrophil infiltration and subsequent tissue damage. The immune response stimulated through treatment with glucan protects the host, not only by increasing its survival rate but also by controlling exacerbated inflammatory response, preventing multiple organ dysfunction syndrome (MODS), highlighting the effectiveness of this natural product as a therapeutic intervention against serious infections.

To clarify the immune mechanisms associated with glucan treatment, the most relevant aspects observed in the studies were summarized (Table 1). Based on the data extracted after reading the selected articles and after analysis of Table 1, a figure was generated in order to illustrate the mechanisms of action of glucans in the immune and inflammatory responses (Figure 2).

According to the selected studies, the modulatory actions of the glucans are mainly due to their abilities to reduce proinflammatory cytokines and the activity of enzymes directly linked to the inflammatory process including myeloperoxidase, preventing multiple organ failure and increasing the survival of septic animals (Figure 2).

## 4. Discussion

*β*-Glucan was the main type of glucan studied among the selected articles (88.2%). *β*-Glucans are polysaccharides widely present in the cell wall structure of various plants and microorganisms, and they have a high affinity for receptors linked to the immune response in mammalians. These macromolecules are formed through the union of smaller monosaccharides, *via* glycosidic bonds, and play a fundamental role in the structure of the cell wall of the organisms in which they are present. Glucans are subject to the action of various enzymes responsible for generating the bonds that join the smaller units of monosaccharides and that participate in the construction and conformation of the polysaccharide [39]. This is an important factor since various studies have described how the chemical structure of glucans is directly related to their biological activity [40].

The characteristic glycosidic bonds of these molecules are related to their origin and bioactivity. The *β*-glucans isolated from fungi have a 1 → 3 bond with branches at 1 → 6, whereas those isolated from cereals, such as wheat, oats, and rice, have bonds at 1 → 3 and 1 → 4 and have long linear chains [41]. The positions of these bonds have a direct effect on the biological activity of *β*-glucans, with the 1 → 3 bond being most associated with strong immune responses, whereas the 1 → 4 bond is associated with a lower or no immune responses [40]. Among the selected articles, six [26, 27, 28, 29, 30, 35] specified the use of (1 → 3)-*β*-glucan with associated increases in IL-10 [26] and IL-6 [26], reductions of TNF-*α* [27, 35] and IL-6 [27, 35], and increased survival rates [27, 30, 34, 35]. These effects prevented cardiac dysfunction and inhibited cardiomyocyte apoptosis [28], reducing TLR4 expression [29], NF-*κ*B activity [29], and cytoplasmic levels of HMGB1 [29], in addition to decreasing myeloid suppressor cells [35]. Meanwhile, two studies [36, 37] reported that 1 → 3-1 → 6-*β*-glucans are associated with a reduction in zinc and copper levels [36] in the liver, lung, kidney, heart, and diaphragm; a reduction in TNF-*α* [37] and MDA [37] levels; and a reduction of neutrophil infiltration in all tissues investigated such as the lung, heart, liver, kidney, diaphragm, and brain [37].

Among the articles included in the review, only one evaluated the activity of *α*-glucans in a model of sepsis [35]. The *α*-glucan YCP isolated from the fungus *Phoma herbarum* showed significant immunomodulatory activities, reducing inflammatory cytokines, such as IL-6 and serum TNF-*α*, in septic mice and regulating the frequency of myeloid suppressor cells [42]. The *α*-glucans synthesized by fungi have complex structures and different glycosidic, and although there are more studies on the bioactivity of *β*-glucans, *α*-glucans isolated from the walls of fungi have shown immunomodulatory activity similar to that of *β*-glucans, as well as antitumor activity after modifications to improve their solubility [43].

Similar to that of other polysaccharides and dietary fibers, the mechanisms underlying the effects of *β*-glucans include increased absorption of nutrients and improvement of the viscosity of intestinal contents. In addition, *β*-glucans might represent a potential source for fermentation by microorganisms in the small intestine, promoting a prebiotic effect [44]. Arena et al. [45] conducted a study evaluating the symbiotic potential between *β*-glucans and probiotic strains. Mixtures of probiotic microorganisms and barley *β*-glucans presented synergistic effects, modulating at transcriptional level, proinflammatory genes encoding TNF-*α*, NF-*κ*B, IL-8, and IL-1*β*. In addition, incubation with probiotics significantly increased IL-10 gene expression, leading to an anti-inflammatory effect.

*β*-Glucans were shown to reduce proinflammatory cytokines, stimulate the release of anti-inflammatory cytokines, and increase the formation of antioxidants [46, 47, 48]. Among the studies analyzed in the review, it was observed that during sepsis, treatment with glucans can reduce inflammatory mediators. With respect to these mediators, of the articles included in this review, 29% reported reductions of TNF-*α* [20, 25, 27, 35, 37], 29% described attenuated levels of IL-6 [20, 25, 27, 31, 35], and 5.8% diminished production of IL-1*β* in glucan-treated septic mice. However, in contrast to the aforementioned data, Babayigit [19] showed an increase in IL-6; however, the administered dose of glucan was 2 mg/kg, i.e., lower than that tested in other studies in which decreased IL-6 was observed, such as the study by Newsome et al. [31] that used a dose of 10 mg/kg. Also, Pan et al. [6] described an increase in TNF-*α* and IL-6 in septic individuals treated with *β*-glucans (dose of 500 *µ*g). The varied results observed for glucans on IL-6 may be due their different doses in the studies. In addition, Murphy et al. [49] showed that *β*-glucans from the same *Lentinus edodes* mushroom, one isolated using hot water extraction and the other of commercial origin, had different effects, specifically reducing inflammatory cytokines and reducing macrophage phagocytic activity after stimulation with LPS. This suggests that the source and extraction methods can influence the results.

In this study, four articles [25, 26, 31, 32] addressed the impact of glucans on IL-10 production and its subsequent effects on sepsis outcome. In one of these [25], increased IL-10 reduced TNF-*α*, IL-6, and IL-1*β* levels which were noted, whereas in Newsome et al. [31], it was obtained reductions in IL-10 and IL-6. The authors showed a reduction in colony-forming unit (CFUs) and an increase in animal survival.

In addition, certain genes, transcription factors, and receptors related to the inflammatory response were analyzed. NF-*κ*B and nuclear factor interleukin 6 (NF-IL6 or CCAAT enhancer binding protein (C/EBP)) are transcriptional activator proteins that participate in the induction of numerous cellular genes and are intrinsically involved in the regulation of inflammatory cytokine genes [50, 51, 52]. NF-*κ*B was evaluated in three (17.6%) of the studies analyzed in this review [27, 29, 35]. In Williams et al. [27], a reduction in nuclear binding activity NF-*κ*B and NF-IL6 in addition to TNF-*α* and IL-6 levels was observed regardless of glucan treatment schedule (pre- and postsepsis treatment). In the studies by Ha et al. [29] and Liu et al. [35], it was demonstrated that glucans increase NF-*κ*B activation. Also, Liu et al. [35] showed decreased TNF-*α* and IL-6 quantities following glucan treatment. These variable results could be related to the different experimental approaches adopted in each study.

The expression of TLR-2 and TLR-4 in patients with sepsis was upregulated compared to that of healthy individuals [53, 54]. In this review, two studies (11.7%) evaluated these receptors; both reported a decrease in the expression of TLR-2 and TLR-4 [29, 30]. In these studies, the treatment protocol, form of administration, and evaluation period were identical, differing only in the dose administered, specifically 40 mg/kg for Ha et al. [29] and 50 mg/kg for Williams et al. [30].

The mechanisms underlying the protective effects of glucans against CLP–induced sepsis are due to an enhanced host immune response against the infection. A key aspect is the modulation of the expression of mortality-related genes, such as the overexpression of TLRs, contributing to the progression of the inflammatory injury cycle during sepsis [17]. Thus, treatment with glucans, by modulating the expression of these genes, attenuated proinflammatory responses through signal transduction pathways mediated by downregulating receptors, which may lead to improved long-term survival. Additionally, glucans activate the phosphoinositide 3-kinase signaling pathway in CLP sepsis; this pathway limits the activation of signaling and the expression of proinflammatory mediators [55, 56].

Another important assay when studying septic conditions is the level of myeloperoxidase (MPO), as it is directly related to neutrophil infiltration and activation. In Babayigit [19], Bedirli [20], and Demir et al. [38], the production of this enzyme was evaluated, and its increase in CLP animals was attenuated by *β*-glucan treatment [19, 20, 38]. Also, MDA, a biomarker of lipid peroxidation induced by oxidative stress and indicative of severe sepsis [57], was described in three studies [25, 37, 38] corresponding to 17.6% of the total; these demonstrated MDA formation is reduced after treatment with glucans.

It is worth mentioning that different routes of administration were observed among the articles selected (intragastric gavage, intraperitoneal, subcutaneous, intramuscular, and intravenous), with the intraperitoneal route being the most used among the articles included [11]. There is no established consensus on the best route of administration of *β*-glucan in septic models. Among the articles selected in this study, the intraperitoneal administration achieved greater animals survival and significant immunomodulatory activity. However, it is worth noting that the subcutaneous route was also one of the main choices for the treatment of septic mice [21, 58, 59, 60], reaching satisfactory beneficial results. Thinking ahead and acceptance in humans, the subcutaneous routes have advantages in that no professional qualification is required for application in contrast to IV and IM administrations, the injections are less painful, the risk of infection is lower with SC injections than with IV injections, and, if it does occur, the infeccion is generally limited to a local infection rather than a systemic infection. In addition, SC injections offer a wider range of alternatives sites than IM injections for patients requiring multiple doses [61, 62]. Another systematic study highlighted patient compliance, with time savings and autonomy for home treatment being some of the reasons for preferring the subcutaneous route of administration [63].

The ability of glucans to act as modulators of the inflammatory response not only reveals their therapeutic potential but also highlights their biotechnological relevance, providing prospects for the medical community in the prognosis of septic individuals. In this context, glucans emerge as a promising alternatives as therapies and adjuvants. In a phase 2 randomized clinical trial evaluating the effect of oral *β*-glucan supplementation, this potential was observed through an increase in IgG in patients who received a vaccine booster associated with *β*-glucan supplementation. The research aimed to improve IgG antibody titers and seroconversion rates, which are associated with improved survival in patients with high-risk neuroblastoma. To do this, the authors selected 107 patients, divided into groups: group 1 did not receive *β*-glucan for the first 5 weeks, and group 2 received an oral regimen of *β*-glucan (40 mg/kg/d for 14 consecutive days and then 14 days without) from week 1. From week 6, all 107 patients received the *β*-glucan regimen for 1 year or until disease progression. Seven subcutaneous vaccine injections were administered (weeks 1, 2, 3, 8, 20, 32, and 52), each consisting of 30 *μ*g of GD2 and 30 *μ*g of GD3. It was observed that by administering *β*-glucan early during the initiation phase (group 2), the IgG1 anti-GD2 antibody response in vaccinated patients was statistically higher than in the control (group 1). In addition, a high antibody response (≥230 ng/mL) at week 8 of the vaccine correlated significantly with improved progression-free survival. The authors also emphasized that treatment with *β*-glucan meets many of the requirements for an effective adjuvant [64]. Vetvicka [65] states that *β*-glucans have been widely used to protect against infections. Using various experimental models, *β*-glucans have been shown to protect against bacterial and protozoan infections, as well as to increase the efficacy of antibiotics in infections caused by antimicrobial-resistant bacteria. In addition, this review has shown its effectiveness in modulating immune response mediators such as cytokines, reducing inflammatory infiltrates and preventing tissue damage, corroborating its crucial role in attenuating the exacerbated inflammatory response characteristic of septic conditions. It is necessary to invest in more *in vivo* studies, especially in a mouse model of lethal sepsis, so that the prospects for the future in the clinics can be confirmed, ensuring the use of glucans in systemic infections.

Long-term adverse effects have been debated to ensure the safety of glucan supplementation. Studies such as that by Cardenas et al. [66] evaluate the toxicity of *β*-glucan extract derived from yeast which was evaluated in 44 patients, and in general, good tolerance was obtained, even at the highest doses. In addition, the 27 patients who survived had no adverse effects after 5 years of treatment. Meng [67] evaluated the ability of *β*-glucan (orally 1 time/day for 12 weeks) to prevent the number of infections in children during the cold season, and even at higher concentrations, no relevant adverse effects were recorded. In the work of Urbancikova et al. [68], the effect of *β*-glucan in the treatment of herpes was observed, and even with the glucan extract being used for 120 consecutive days, no adverse effects were observed. Therefore, the evidence suggests that the use of extracts containing *β*-glucans has not caused any adverse effects.

Sepsis is a very complex disease which depends on inflammatory and anti-inflammatory pathways to comprise effective immune responses as well as accomplish homeostasis. The intricate network of players and pathways underlying sepsis fatality or recovery means that disease management depends on a timely and appropriate management, which therefore reflect on its outcome. It is important to highlight that sepsis control goes beyond a single compound given at a unique time point, such as the importance of rehydration, hemodynamic support, antibiotic administration, and control of the source of infection, among other measures [69]. This is specially important when nutritional ingredients such as glucans can be taken daily way before and even during the course of sepsis, greatly influencing disease evolution. This means that a successful treatment all depends on a series of factors which include therapy and patients immune background. Nonetheless, glucans as other natural products are powerful resources to be individually considered to sepsis and other mechanistically similar diseases.

Considering the complexity of sepsis, as well as the continuous search for novel effective therapies, glucans have emerged as promising threrapies or adjuvants for conventional treatments. The potential of glucans is evidenced by their ability to modulate the immune response and reduce tissue damage. Glucans therefore appear to be a promising biotechnological alternative for sepsis management.

## Figures and Tables

**Table 1:** Distribution of selected studies according to formulation, type of study, type and dose/concentration of the glucan, route of administration, treatment schedule, immunological activity, and references.

Source	Glucan type	Type of study	Dosage/concentration	Route of administration	Treatment schedule	Immunological activity	Ref.
Microorganism: yeast, *Saccharomyces cerevisiae*	*β*-Glucan (microparticulate form, suspended with saline, 50 mg kg^−1^, Immunex R, M.N.C.)	*In vivo* (*rats: Wistar albino rats of both sexes*)	50 mg (kg)	GIG	One time day/for 10 days + 30 min before CLP	↓ TNF-*α*, ↓ IL-6, ↑ IL-10 soro ↑ superoxide dismutase (SOD), and catalase (CAT) in the liver tissue ↓ malondialdehyde (MDA) in the liver	[25]

Plant: cereal and barley	*β*-D-Glucan (Sigma, St. Louis, MO, USA)	*In vivo* (*rats: male Wistar albino*)	2 mg (kg)	IP	One time post-CLP surgery	↓ Pulmonary MPO; ↑ leukocytes in the blood, ↑ monocytes in the blood; ↓ neutrophils in the blood; ↓ neutrophils and lymphocytes in the BAL, ↑ monocytes in the BAL; ↑ serum IL-6, ↓ alveolar hemorrhage	[19]

Plant: cereal and barley	*β*-D-Glucan (Sigma Chemical Company, St Louis, MO)	*In vivo* (*rats: male Wistar*)	2 mg (kg)	IP	Just after CLP with an additional one injection at 4 hr after CLP	↑ Survival rate to 63% ↓ the levels of TNF-*α*, IL-1*β*, and IL-6 ↓ lung MPO ↓ lung ICAM-1	[20]

Not specified	Pachyman (Associates of Cape Cod, Falmouth, MA, USA)	*In vivo* (*mice: female FcGRIIb −/− C57BL/6*)	50 mg (kg)	IV	3 and 6 hr post-CLP surgery	↑ IL-10 ↑ IL-6	[26]

Microorganism: yeast, *S. cerevisiae*, and fungi, *Sclerotium glucanicum*	(1 → 3)-*β*-D-Glucans (glucan phosphate and scleroglucan)	*In vivo* (*mice: male ICR/HSD*)	50 mg (kg)	IP	Pretreatment 1 hr before CLP and posttreatment 15 min after CLP	Pretreatment: glucan phosphate: ↓ liver NF-*κ*B and NF-IL6; ↓ lung NF-*κ*B and NF-IL6; ↑ survival rate by 65%. ↓ hepatic TNF-*α* and IL-6; ↓ lung TNF-*α* and IL-6; scleroglucan: ↓ liver NF-*κ*B and NF-IL6; ↓ lung NF-*κ*B and NF-IL6; ↑ increases survival rate in 75% posttreatment: glucan phosphate: ↓ liver NF-*κ*B and NF-IL6; ↓ lung NF-*κ*B and NF-IL6; ↑ survival rate by 65%; ↓ hepatic TNF-*α*; ↓ lung TNF-*α* and IL-6	[27]

Microorganism: yeast, *S. cerevisiae*	(1 → 3)-D-Glucan (glucan phosphate)	*In vivo* (*mice: male ICR/HSD*)	40 mg (kg)	IP	1 hr before induction of CLP	↓ Cardiac dysfunction activates the phosphoinositide 3-kinase/Akt pathway; ↓ myocardial MIF expression; ↓ cardiomyocyte apoptosis; prevented the decrease phospho-Akt and phospho-GSK-3*β*; prevented the decrease in Bcl-2	[28]

Microorganism: yeast, *S. cerevisiae*	(1 → 3)-D-Glucan (glucan phosphate)	*In vivo* (*mice: male ICR/HSD*)	40 mg (kg)	IP	1 hr before induction of CLP (GP foi dissolvido em solução salina)	↓ Translocation of HMGB1 ↓TLR4 expression ↓ myocardium NF-*κ*B	[29]

Microorganism: yeast, *S. cerevisiae*	(1 → 3)-D-Glucan (glucan phosphate)	*In vivo* (*mice: male ICR/HSD*)	50 mg (kg)	IP	1 hr before induction of CLP	↑ Long-term survival (20% vs. 70%) ↓ TLR2/4 gene and TLR4 protein expression	[30]

Microorganism: nonrecombinant yeast strain, *S. cerevisiae*	*β*-Glucan PGG glucan (Imprime-PGG) (Eagan, MN)	*In vivo* (*mice: male*, *female*, *and ovariectomized female CD-1 mice*)	10 mg (kg)	IP	1 hr after induction of CLP	Male: ↑ survival only 24 hr femal: ↑ survival over a 10-day period, ↓ interleukin-6 (IL-6) and IL-10 ↓ CFU in the liver, ovariectomy: abrogated the response to PGG glucan	[31]

Microorganism: yeast, *S. cerevisiae*	(1 → 3)-D-Glucan (glucan phosphate)	*In vivo* (*mice: male ICR/HSD*)	40 mg (kg)	IP	1 hr before induction of CLP	↑ Survival by 60%; ↑ PI3K activity	[32]

Plant: cereal and barley	*β*-D-Glucan (Sigma Chemical, St. Louis, MO)	*In vivo* (*rats: male Wistar*)	2 mg (kg)	IM	After induction of CLP	↓ Weight loss; ↓ cumulative adhesion score	[33]

Not specified	Glucan-P (Accurate Chemical and Scientific Corp., Westbury, NY) and glucan-F (Laboratory of the Late Dr. N. R. DiLuzio. New Orleans, LA)	*In vivo* (*rats: male Sprague–Dawley*)	10 mg (kg)	IV	Daily for 5 consecutive days	↑ Survival 7-day (glucan-P and/or glucan-F + ampicillin)	[34]

Microorganism: fungi, *P. herbarum*	*α*-Glucan (YCP) (phoma herbarum YS4108 fungi)	*In vivo* (*mice: male c57bl/6j*)	20 mg (kg)	IP	2 hr before, 4 hr after, and 24 hr after CLP	YCP: ↑ survival from 39% to 72% on d 10 post-CLP ↓ myeloid-derived suppressor cells (MDSCs) in the lungs and livers ↓ STAT3 pathway activation ↑ Interferon regulatory factor-8 (IRF-8) ↓ IL-6 e TNF-*α* when BM-derived MDSCs were co-cultured with T cells, YCP dose-dependently ↑ Arg-1/iNOS, and activated the NF-*κ*B pathway	[35]

Microorganism: yeast, *S. cerevisiae*	1 → 3−1 → 6-*β*-D-Glucan (Mustafa Nevzat Company, Turkey. *S. cerevisiae*)	*In vivo* (*rats: albino wistar*)	50 mg (kg)	GIG	Once a day for 10 days	↓ Zinc and copper levels of the liver, lung, kidney, heart, and diaphragm. ↓ Tissue damage	[36]

Microorganism—yeast*—S. cerevisiae*	1 → 3−1 → 6-*β*-D-glucan (Mustafa Nevzat Company, Turkey. *S. cerevisiae*)	*In vivo* (*rats: Wistar albino rats of both sexes*)	50 mg (kg)	GIG	Once daily for 10 days and 30 min prior to and 6 hr after the CLP	↓TNF-*α* ↓The MDA levels in the liver, kidney, heart, lung, diaphragm, and brain reversed the GSH level ↓ neutrophil infiltration	[37]

Plant: cereal and barley	*β*-glucan (CAS: 9041-22-9) Sigma (Shanghai, China)	*In vitro* (*raw 246.7*) *in vivo* (*mice, female ICR*)	30 *μ*g (mL) (in vitro) 500 *μ*g (in vivo)	IP	Twice a week before sacrifice	↓ Lung and liver injury ↓ CFU blood and peritoneum (*β*-glucan and *β*-glucan + SPIO); ↓ inflammatory cell infiltration and hyperemia in the alveolar walls (*β*-glucan, *β*-glucan + SPIO); ↓ vacuolization and sinusoidal congestion in the liver (*β*-glucano + SPIO); ↑ TNF-*α*, IL-1*β*, and IL-6 mRNA expression	[6]

Plant: cereal and barley	*β*-Glucan (*β*-D-glucan, Sigma, St. Louis, MO, USA)	*In vivo* (*rats*)	4 mg (kg)	IP	Following CLP (a) single dose of 4 mg *β*-glucan (kg)	↓ Plasma MPO/AOPP/MDA ↓ lung MDA ↓ liver MDA	[38]

GIG, intragastric gavage; IP, intraperitonealy; IM, intramuscularly; IV, intravenously; CLP, cecal ligation and puncture; BAL, bronchoalveolar lavage; MIF, migration inhibitory factor; ICAM, intercellular adhesion molecule; MPO, myeloperoxidase; Ref, references.

## Data Availability

The article data supporting this review are from previously reported studies and datasets, which have been cited. The processed data are available from the corresponding author upon request.
